# Constructing Counting and Arithmetic Learning Trajectories for Kindergarteners: A Preliminary Investigation in Taiwan

**DOI:** 10.3390/children9121994

**Published:** 2022-12-19

**Authors:** Chung-Chin Wu

**Affiliations:** Department of Early Childhood Education, National Pingtung University, Pingtung 900391, Taiwan; minin72704@mail.nptu.edu.tw

**Keywords:** arithmetic, counting, developmental progression, kindergartener, learning trajectory

## Abstract

Mathematics learning trajectories (LTs) for students above elementary school level are widely investigated. Recently, LTs for kindergarteners have also attracted attention, but in those studies the LTs were based on Western samples, and it is unclear whether they also involved culture and gender differences. Therefore, the purposes of this study were twofold: (1) construct a counting and arithmetic LT based on an Eastern sample and (2) show its similarities and differences by gender. The constructed LT contains 13 hypothesized levels of mathematical concepts according to previous research, and 59 kindergarteners (26 boys and 33 girls) participated in this study and completed a counting and arithmetic test to examine empirically the theoretical LT. The results showed that empirically, there were eight and nine conceptual levels for boys and girls, respectively, and boys and girls mastered concepts in a similar order (basic arithmetic→basic counting→advanced counting→mediocre arithmetic→advanced arithmetic), with the first part differing from the hypothesized LT. Within this developmental progression, girls showed a different path from advanced counting to mediocre arithmetic. The findings show gender and culture differences for the LTs for kindergarteners, which contradicts most previous research based on Western samples.

## 1. Introduction

The competence of kindergarteners in mathematics is a critical determinant of their later success in not only mathematics but also literacy and science [1,2,3]. However, early mathematics has long been regarded as unimportant learning content because kindergarten teachers often have negative experiences about mathematics and lack knowledge about the topics that can be taught [4]. Consequently, most kindergarteners are repeatedly taught basic mathematical content (e.g., verbal counting from 1 to 5) that they have already mastered, and this form of instruction has been shown to have negative effects on later mathematical performance. By contrast, teaching advanced mathematical content has been shown to have positive effects on later mathematical and reading performance [5,6]. Therefore, to improve the teaching and learning of mathematics, it is important to provide kindergarten teachers with a conceptual map that indicates clearly the levels of difficulty in mathematical content.

Recently, Li et al. reported how two children who scored the same but with different response patterns on a mathematical problem-solving test before intervention, then showed individual differences in their developmental progression during the intervention period [7]. However, in that study, the authors used only the response patterns of two kindergarteners of unknown gender, and they did not introduce an analysis method to enable their findings on the developmental progression to be generalized. It remains unclear how different kindergarteners of different gender learn the same advanced mathematical concepts through different developmental progressions. In practice, it is more important for teachers to identify kindergarteners’ mastery levels and developmental progressions in mathematical concepts, and to further structure teaching activities corresponding to kindergarteners’ current level of mathematical thinking or one level above to help individual them master advanced mathematical concepts [8].

Developmental progression in mathematical concepts is also called a learning trajectory (LT). An LT is a developmental mapping of mathematical thinking and concepts that contains the structure of the mathematical concepts and the developmental levels of thinking; it describes which concepts are used to solve problems and how learners think in increasingly sophisticated ways. It is important to note that the levels in an LT are averages for the mathematical concepts or skills that most kindergarteners have developed [9,10]. Various efforts have been made to construct theoretical LTs for mathematical education and then use them in practice. However, most LTs in previous studies were built solely on a theoretical level and based on participants above the first grade [11]. For example, Confrey et al. tried to organize a mathematical LT for equipartitioning from K-8 based on the Common Core State Standards for Mathematics [12]. However, the learning content and materials in kindergartens differ largely from those above the first grade, suggesting that a mathematical LT for kindergarteners may play a special role that makes it different from other grades. Consequently, there has been little research on counting and arithmetic LTs for kindergarteners.

To date, it seems that the only relevant work is that by Clements et al., who proposed two hypothesized LTs for kindergarteners’ counting and arithmetic, and they argued that that arithmetic LT is interwoven with the counting LT [9]. This suggests that there may be only one LT comprising both counting and arithmetic concepts. However, it is yet to be shown empirically how these counting and arithmetic concepts develop sequentially. According to research on the development of counting and arithmetic, counting develops before arithmetic and is fundamental to the latter. In counting and arithmetic LTs, the level-1 concept is verbal counting (i.e., counting out number words) and the level-2 concept is one-to-one correspondence counting (i.e., counting out number words and point to corresponding objects in order). Sequentially, level-3 developmental progression is to acquire the cardinal principle (i.e., to know that the last counting number represents the total amount of objects). Level-4 developmental progression is endorsed numeral knowledge that enables kindergarteners to identify written numerals and connect them with number words. Level-5 developmental progression is finding the sum of two numbers. Level-6 developmental progression is being able to find the difference (under 10) of two numbers. Level-7 developmental progression is being able to compose and decompose numbers. Level-8 and level-9 developmental progression is being able to count forward and backward, respectively. Level-10 developmental progression is being able to find an unknown addend. Level-11 developmental progression is being able to find an unknown summand [2,3,9,13,14,15,16]. It is interesting that unknown-subtrahend and unknown-minuend questions are not included in the hypothesized LTs, perhaps because they are difficult for kindergarteners in Western countries. However, kindergarteners have shown that they can understand and solve both unknown-subtrahend and unknown-minuend questions [17,18]. According to the above logical arrangement of the hypothesized LT, it is reasonable to hypothesize that being to solve unknown-subtrahend and unknown-minuend problems corresponds to level-12 and level-13 developmental progressions. In summary, the hypothesized counting-and-learning LT contains 13 levels of developmental progression, and the development corresponds to the following order of five stages: basic counting→basic arithmetic→advanced counting→mediocre arithmetic→advanced arithmetic. Under investigation is whether this theoretical LT with 13 levels in five ordered stages can be supported by empirical observations.

A literature review showed that in previous studies, LTs were constructed mostly based on Western samples, with few kindergarteners in Eastern countries included. Several studies and a meta-analysis have shown that there are gender and national differences in mathematical achievement [19], suggesting that counting and arithmetic LTs may also differ across gender and culture. It remains unclear whether there are gender and cultural differences in counting and arithmetic LTs for kindergarteners based on Eastern participants and empirical analysis.

The objective of the study reported herein was twofold:

- to clarify the differences between the theoretical and empirical LTs of counting and arithmetic concepts.
- to examine the differences between boys and girls regarding the LT of counting and arithmetic concepts.

## 2. Methodology

### 2.1. Participants

Two-stage sampling was used to select participants. In the first stage, two kindergartens in Taiwan were randomly selected, and two classes were consequentially selected from each of these two kindergartens. The parents of 59 kindergarteners allowed them to participate in this study, and the children were 26 boys and 33 girls aged 5.

### 2.2. Instruments

A Chinese version of a counting and arithmetic concept test for kindergarteners (C-CAAT) was developed with reference to part of the commonly used test of early mathematics concept (TEMA-3) developed by Ginsburg and Baroody. TEMA-3 was a standardized test, and it was developed to assess mathematics performance for 3- to 8-year-old children. It contained several subtests with a total of 72 items designed to measure subitizing, counting, number properties, quantity comparison, addition, subtraction, multiplication, and division [20]. TEMA-3 is considered to be a reliable instrument (all internal consistency reliabilities and test–retest reliabilities are above 0.92 and 0.80, respectively) for measuring mathematical concepts in children between the ages of 0 and 8 [21]. Recently, researchers applied a Rasch model to clarify the psychometric properties of TEMA-3, and it was demonstrated to be a valid instrument with good technical qualities, interpretable internal structure among items, and reasonable convergent validity [22].

C-CAAT contains 40 items to test kindergarteners’ counting and arithmetic concepts, including oral counting from 1 to 30 (one item), one-to-one correspondence counting within 30 (three items, e.g., “Please tell me how many apples are here”), cardinality within 30 (three items, e.g., “Please tell me how many apples are here in total”), numerical literacy within 30 (three items, e.g., “Please point out the number 10 from these number cards), sum (under 10) of two numbers (two items, e.g., “Mother has five apples and father gives her another two apples. How many apples does mother have now?”), difference (under 10) between two numbers (four items, e.g., “Mother has five apples, two of which are then eaten by your younger brother. How many apples does mother have now?”), compose and decompose number 10 (two items, e.g., “There are 10 apples here. Please put them into two baskets”), forward verbal counting 30 numbers from a specific number within 30 (three items, e.g., counting forward from the number 8), backward verbal counting from a specific number within 30 to 1 (three items, e.g., counting backward from the number 21), unknown addend (within 30) (four items, e.g., “Mother brings five apples home, but father says that nine apples are enough for eating. How many apples should mother buy to make nine?”), unknown subtrahend (within 30) (four items), unknown summand (within 30) (four items), and unknown minuend (within 30) (four items). The internal consistency reliabilities of all items in the C-CAAT are above 0.90. Because of their limited mental arithmetic, the kindergarteners were allowed to use subitizing or objects (e.g., 3D-printed apples) to solve most of the counting problems (except for those related to verbal counting) and all the arithmetic problems. A completely correct answer to a question was given a score of 1 to represent that the answering kindergartener had either truly mastered or had completely developed the concept being tested, while any incomplete or incorrect answer was given a score of 0. To prevent the kindergarteners from guessing and therefore misleading our understanding of their grasp of the tested concept, an average score was calculated to form a single indicator to identify whether a kindergartener had mastered a specific concept, with complete mastery associated with only an average score of 1. Finally, there are 13 indicators representing 13 mathematical concepts, with indicators 1–4, 7, 8, and 9 related to counting concepts and indicators 5, 6, and 10–13 related to arithmetic concepts.

### 2.3. Analysis

To construct an LT of counting and arithmetic, the degree of difficulty (represented by the fraction of correct answers, hereinafter referred to as the correct rate) for each item was calculated first to identify the levels among the concepts (the structure of the mathematical concepts). The correct rate for each item was calculated by dividing the number of correct answers by the number of all answers for that item; the higher (resp. lower) the correct rate, the easier (resp. harder) the concept. In turn, order theory was introduced to illustrate the developmental progression (LT) from concept to concept.

Bart and Krus proposed order theory to identify prerequisites and mutual relationships among concepts as well as the transition relation from concept to concept [23]. The logical prerequisite relation is identified by examining the magnitudes of the frequencies in 2 × 2 cross-tabulation. An exemplar for the rationale of order theory is given in Table 1, where there are two items i and j, and a correct answer to item i is the logical prerequisite for success on item j. Four possible response patterns are formed: response pattern (11) [resp. (00)] represents the participant having answered items i and j correctly (resp. incorrectly); response pattern (10) represents the participant having answered item i correctly but item j incorrectly; response pattern (01) represents the participant having answered item i incorrectly, but item j correctly. Theoretically, response patterns (11), (00), and (10) are reasonable, because the participant is expected to answer both items correctly or incorrectly or just answer the easier item correctly; these are known as confirmatory responses. By contrast, response (01) is unreasonable, because the participant is not expected to master a concept or develop it well in the absence of its prerequisite; this is known as a disconfirmatory response. In Table 1, A, B, C, and D stand for the frequencies of response patterns (11), (10), (01), and (00), respectively, and N represents the total frequency (equal to the sum over all participants). C/N represents the probability of a disconfirmatory response. The prerequisite relation of item i to item j is confirmed if C/N is less than or equal to the tolerance level, whereas items i and j are independent if C/N is greater than the tolerance level. Also, a mutual relation between items i and j is established if there are prerequisite relations from item i to item j and from item j to item i. There is a transition relation if there are two prerequisite relations, one from item i to item j and another from item j to item k, whereupon these two prerequisite relations are replaced by a transition relation from item i to item k. The range of the tolerance level is from 0 to 1, and it was set at 0.10 in this study according to previous research [24]. The probability of a disconfirmatory response between any two items is presented in a k × k matrix table (where k is the total number of items).

## 3. Results

### 3.1. Levels of Counting and Arithmetic Concepts in Learning Trajectory

**Figure 1:**
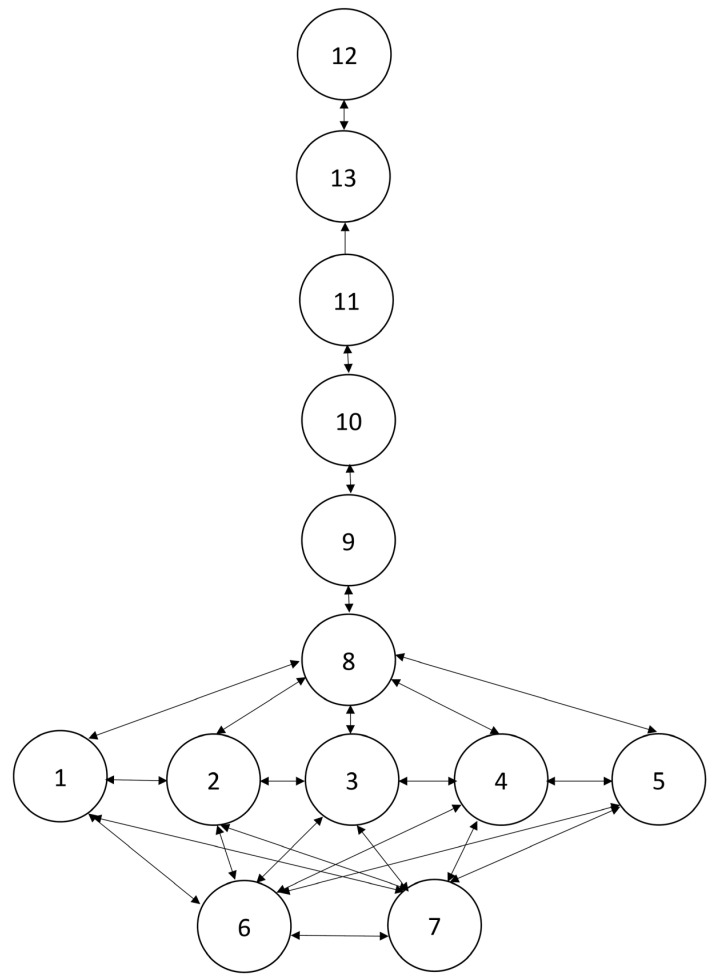
Learning trajectory of counting and arithmetic concepts for boys. Notes: 1 = oral counting from 1 to 30; 2 = one-to-one correspondence counting within 30; 3 = cardinality within 30; 4 = numerical literacy within 30; 5 = sum (under 10) of two numbers; 6 = difference (under 10) between two numbers; 7 = compose and decompose number 10; 8 = forward verbal counting 30 numbers from a specific number within 30; 9 = backward verbal counting from a specific number within 30 to 1; 10 = unknown addend (within 30); 11 = unknown subtrahend (within 30); 12 = unknown summand (within 30); 13 = unknown minuend (within 30).

According to the correct rates (CRs), the counting and arithmetic LTs for boys and girls have eight and nine levels, respectively. For boys, the level-1 concepts are “difference (under 10) between two numbers” and “compose and decompose number 10” (columns 6 and 7, respectively, in Table 2; the CR is 98.31%). The level-2 concepts are “oral counting from 1 to 30,” “one-to-one correspondence counting within 30,” “cardinality within 30,” “numerical literacy within 30,” and “sum (under 10) of two numbers” (columns 1–5, respectively, in Table 2; the CR is 96.61%). The level-3 and level-4 concepts are “forward verbal counting 30 numbers from a specific number within 30” and “backward verbal counting from a specific number within 30 to 1,” respectively (columns 8 and 9, respectively, in Table 2; the CRs are 88.14% and 84.75%). The level-5 and level-6 concepts are “unknown addend (within 30)” and “unknown subtrahend (within 30),” respectively (columns 10 and 11, respectively, in Table 2; the CRs are 81.36% and 76.27%). The level-7 and level-8 concepts are “unknown minuend (within 30)” and “unknown summand (within 30),” respectively (columns 13 and 12, respectively, in Table 2; the CRs are 69.49% and 61.02%). This structure is shown in Figure 1.

**Figure 2:**
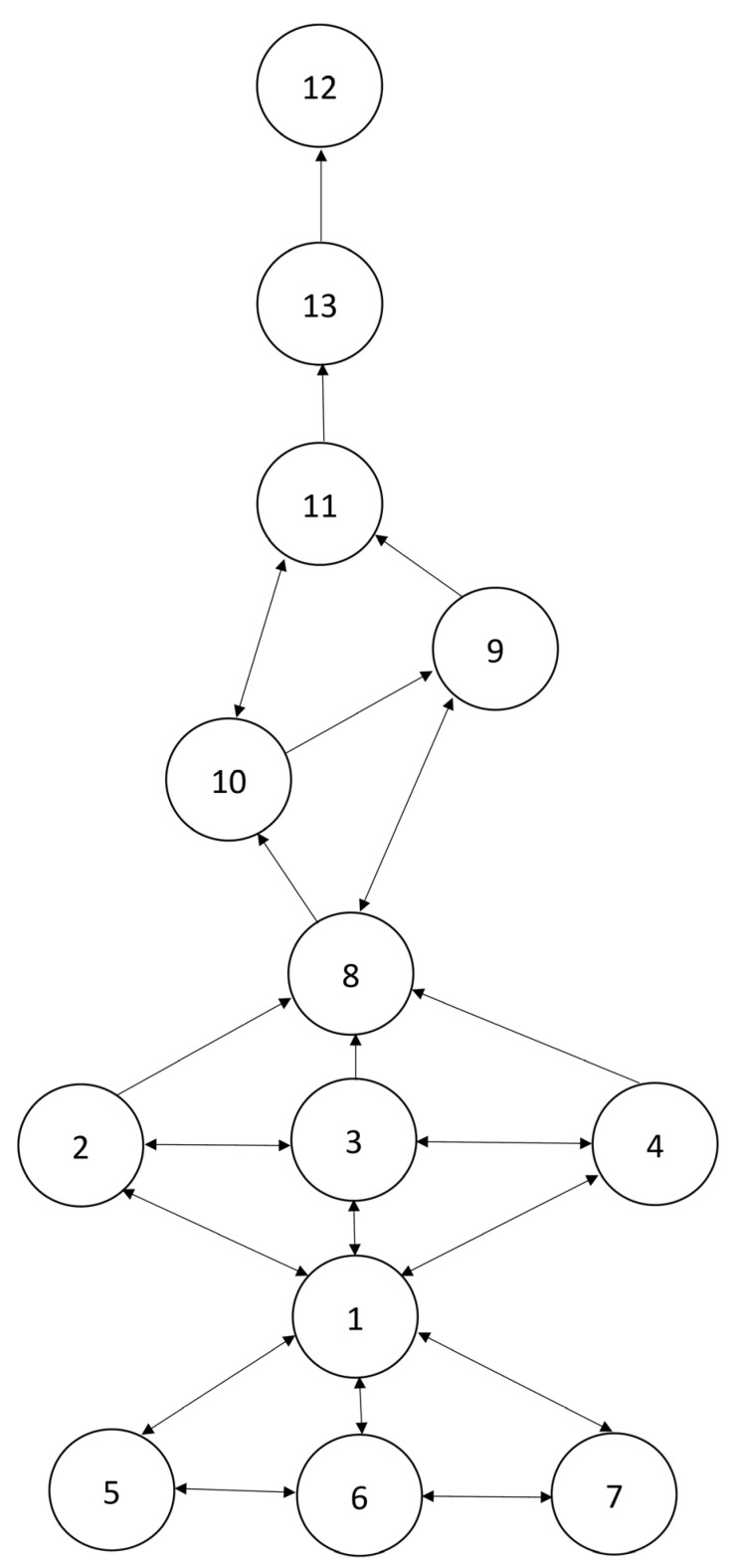
Learning trajectory of counting and arithmetic concepts for girls. Notes: 1 = oral counting from 1 to 30; 2 = one-to-one correspondence counting within 30; 3 = cardinality within 30; 4 = numerical literacy within 30; 5 = sum (under 10) of two numbers; 6 = difference (under 10) between two numbers; 7 = compose and decompose number 10; 8 = forward verbal counting 30 numbers from a specific number within 30; 9 = backward verbal counting from a specific number within 30 to 1; 10 = unknown addend (within 30); 11 = unknown subtrahend (within 30); 12 = unknown summand (within 30); 13 = unknown minuend (within 30).

For girls, the level-1 concepts are “sum (under 10) of two numbers,” “difference (under 10) between two numbers,” and “compose and decompose number 10” (the CR is 100%). The level-2 concept is “oral counting from 1 to 30” (the CR is 98.31%). The level-3 concepts are “one-to-one correspondence counting within 30,” “cardinality within 30,” and “numerical literacy within 30” (the CR is 96.61%). The level-4, level-5, and level-6 concepts are “forward verbal counting 30 numbers from a specific number within 30,” “unknown addend (within 30),” and “backward verbal counting from a specific number within 30 to 1,” respectively (the CRs are 84.75%, 83.05%, and 81.36%). The level-7, level-8, and level-9 concepts are “unknown subtrahend (within 30),” “unknown minuend (within 30),” and “unknown summand (within 30),” respectively (the CRs are 76.27%, 64.61%, and 54.24%). This structure is shown in Figure 2.

### 3.2. Learning Trajectories of Counting and Arithmetic Concepts for Boys and Girls

According to the probabilities of disconfirmatory responses for boys in Table 2 and its graphical representation in Figure 1, there is a mutual relation between the level-1 concepts “difference (under 10) between two numbers” and “compose and decompose number 10” (the visualized relations can also be seen in Figure 1, and they are coded as 6 and 7, respectively) and between any two level-2 concepts (“oral counting from 1 to 30,” “one-to-one correspondence counting within 30,” “cardinality within 30,” “numerical literacy within 30,” and “sum (under 10) of two numbers”) (they are coded as 1–5, respectively, in Figure 1). Similarly, there are also mutual relations between all level-2 and all level-1 concepts (6↔1, 6↔2, 6↔3, 6↔4, 6↔5, 7↔1, 7↔2, 7↔4, and 7↔5). The level-3 concept “forward verbal counting 30 numbers from a specific number within 30” shows mutual relations with all level-2 concepts (1↔8, 2↔8, 3↔8, 4↔8, and 5↔8). The level-4 concept “backward verbal counting from a specific number within 30 to 1” also has a mutual relation with the level-3 concept (8↔9). The level-5 concept “unknown addend (within 30)” has a mutual relation with the level-4 concept (9↔10). The level-6 concept “unknown subtrahend (within 30)” also has a mutual relation with the level-5 concept (10↔11). However, there are only prerequisite relations from the level-6 concept to the level-7 concept (“unknown minuend (within 30)”) (11→13), and from the level-7 concept to the level-8 concept (“unknown summand (within 30)”) (13→12).

For girls, there are mutual relations among three level-1 concepts: “sum (above 10) of two numbers,” “difference (under 10) between two numbers,” and “compose and decompose number 10” (5↔6↔7). The level-2 concept “oral counting from 1 to 30” and all level-1 concepts also have mutual relations (5↔1, 6↔1, and 7↔1). Similarly, there are also mutual relations among the level-3 concepts “one-to-one correspondence counting within 30,” “cardinality within 30,” and “numerical literacy within 30” (2↔3↔4), and they have mutual relations with the level-2 concept (1→2, 1→3, and 1→4). In addition, there are prerequisite relations from any one of the three level-3 concepts to the level-4 concept “forward verbal counting 30 numbers from a specific number within 30” (2→8, 3→8, and 4→8). The level-4 concept is a prerequisite for the development of the level-5 concept “unknown addend (within 30)” (8→10), and it has a mutual relation with the level-6 concept “backward verbal counting from 30 to 1” (8↔9). There is a mutual relation from the level-5 concept to the level-7 concept “unknown subtrahend (within 30)” (10↔11), whereas the level-6 concept is a prerequisite for the level-7 concept “unknown subtrahend (within 30)” (9→11). There is also a prerequisite relation from the level-7 concept to the level-8 concept “unknown minuend (within 30)” (11→13), and in turn from the level-8 concept to the level-9 concept “unknown summand (within 30)” (13→12).

## 4. Discussion

Some differences can be found in the structure of the mathematical concepts when comparing the hypothesized LT with the empirical LT. According to the literature review, it was hypothesized that there are 13 levels in the counting and arithmetic LT for kindergarteners. However, there are only 10 levels to be found for the whole sample, and the levels differ across gender: eight levels for boys and nine levels for girls. Both samples showed similar developmental progression with few exceptions in the learning path of the LT.

In the hypothesized LT, the mathematical concepts are assumed to develop in the following order: basic counting concepts (including numbers 1–4 in Figure 1 and Figure 2) (i.e., “oral counting from 1 to 30” and “one-to-one correspondence counting within 30”)→basic arithmetic concepts (including numbers 5–7) (i.e., “sum of two numbers” and “difference of two numbers”)→advanced counting concepts (including numbers 8 and 9)→mediocre arithmetic concepts (including numbers 10 and 11)→advanced arithmetic concepts (including numbers 12 and 13), and these concepts are hypothesized to develop in number order. However, it was shown in both samples that the basic arithmetic concepts are well developed before the progression of basic counting concepts, and the basic counting concepts are well developed before the progression of advanced counting concepts. The mediocre arithmetic concepts were generally mastered after the development of the basic counting concepts. This lower part of the empirical LT (basic arithmetic concepts→basic counting concepts→advanced counting concepts) differs from the predictions based on studies conducted in Western countries [2,9,13,14,15,16]. Nevertheless, it shows that counting and arithmetic concepts are interwoven with each other, as was argued by Clements et al. [9]. However, they did not indicate clearly which arithmetic concepts may be interwoven with which counting concepts and in what order. The present study constructed an LT incorporating counting and arithmetic concepts based on empirical observations, and it indicated clearly that basic knowledge and skills about numbers (e.g., knowledge about numbers within 30 and verbal counting from 1 to 30) are critical for the development of some basic arithmetic concepts (e.g., knowing the sum and difference of two numbers within 10). This may imply that basic counting concepts involving abstract counting over a certain small number (i.e., number 10) without using objects are more difficult for kindergarteners than solving arithmetic problems by using objects. It may also reflect a difference in the structure of mathematical concepts for kindergarteners in different cultures (e.g., Western vs. Eastern). Regarding implications for practice, it may reflect the fact that very basic counting skills (e.g., verbal counting from 1 to 10) are taught repeatedly and are internalized for automatic use in solving basic arithmetic problems.

In addition, the developmental progression from the mastery of mediocre arithmetic concepts to the development of advanced arithmetic concepts basically corresponds to previous findings [17,18]. This shows that the development of the “unknown subtrahend (within 30)” concept is a prerequisite for solving “unknown minuend (within 30)” problems. In turn, the development of the “unknown minuend (within 30)” concept is a prerequisite for solving “unknown summand (within 30)” problems. However, the last developmental progression arises unexpectedly in the opposite direction to that hypothesized in the present study.

Also, there is a major difference by gender. On the common basis of the above basic developmental progression (basic arithmetic→basic counting), boys learned to solve “unknown subtrahend (within 30)” problems through the developmental progression that starts from the first advanced counting skill of “forward verbal counting 30 numbers from a specific number within 30” to the second advanced counting skill of “backward verbal counting from a specific number within 30 to 1” and further achieved through the mediocre arithmetic concept of “unknown addend (within 30).” By contrast, girls learned to solve “unknown subtrahend (within 30)” problems through a different path that starts from mastery of the first advanced counting skill to the developmental progression of the first mediocre counting concept of “unknown addend (within 30).”

## 5. Conclusions

This study has shown that the structure of mathematical concepts for kindergarteners may differ largely between Western and Eastern countries. However, studies that use samples at the same age from different countries with different cultures and large representative samples are clearly needed to clarify the generalizability of the present findings. Regarding the developmental progression of mathematical concepts by gender, the upper and lower parts in the LTs for boys and girls show similar developmental progression. One path is from basic arithmetic concepts to basic counting concepts, the other is from mediocre arithmetic concepts to advanced arithmetic concepts. The primary gender difference in the LT is that girls can use either basic counting concepts (e.g., one-to-one correspondence counting) or the backward counting strategy to solve “unknown addend (within 30)” problems. By contrast, boys may have difficulty in solving mediocre arithmetic problems if their advanced counting concepts are not well developed. Kindergarten teachers are encouraged to design different learning activities corresponding to boys’ and girls’ developmental progression to help kindergarteners master advanced mathematical concepts.

## Figures and Tables

**Table 1:** Exemplar for rationale of order theory.

		Item j		
		Correct	Incorrect	Total
Item i	Correct	A (11)	B (10)	A + B
	Incorrect	C (01)	D (00)	C + D
	Total	A + C	B + D	N

Notes: A, B, C, and D represent frequencies. The numbers in parentheses in the four middle cells represent the response pattern, with the first (resp. second) digit standing for the response to item *i* (resp. *j*) (0: incorrect answer; 1: correct answer).

**Table 2:** Probabilities of disconfirmatory responses for counting and arithmetic concepts by gender.

	1	2	3	4	5	6	7	8	9	10	11	12	13
1	-	0.02/0.00	0.02/0.00	0.02/0.00	0.03/0.02	0.02/0.02	0.03/0.02	0.02/0.00	0.00/0.00	0.00/0.02	0.00/0.02	0.00/0.00	0.00/0.00
2	0.02/0.02	-	0.00/0.00	0.00/0.00	0.03/0.03	0.02/0.03	0.03/0.03	0.00/0.00	0.00/0.02	0.00/0.02	0.00/0.02	0.00/0.00	0.02/0.00
3	0.02/0.02	0.00/0.00	-	0.00/0.00	0.03/0.03	0.02/0.03	0.03/0.03	0.00/0.00	0.00/0.02	0.00/0.02	0.00/0.02	0.00/0.00	0.02/0.00
4	0.02/0.02	0.00/0.00	0.00/0.00	-	0.03/0.03	0.02/0.03	0.03/0.03	0.00/0.00	0.00/0.02	0.00/0.02	0.00/0.02	0.00/0.00	0.02/0.00
5	0.03/0.00	0.03/0.00	0.03/0.00	0.03/0.00	-	0.03/0.00	0.03/0.00	0.03/0.00	0.02/0.00	0.02/0.00	0.02/0.00	0.00/0.00	0.00/0.00
6	0.00/0.00	0.00/0.00	0.00/0.00	0.00/0.00	0.02/0.00	-	0.02/0.00	0.00/0.00	0.00/0.00	0.00/0.00	0.00/0.00	0.00/0.00	0.00/0.00
7	0.02/0.00	0.02/0.00	0.02/0.00	0.02/0.00	0.02/0.00	0.02/0.00	-	0.00/0.00	0.00/0.00	0.00/0.00	0.00/0.00	0.00/0.00	0.00/0.00
8	0.10/0.14	0.08/0.12	0.08/0.12	0.08/0.12	0.12/0.15	0.10/0.15	0.10/0.15	-	0.05/0.03	0.05/0.10	0.05/0.08	0.02/0.00	0.02/0.03
9	0.12/0.17	0.12/0.17	0.12/0.17	0.12/0.17	0.14/0.19	0.14/0.19	0.14/0.19	0.08/0.07	-	0.03/0.12	0.02/0.08	0.02/0.00	0.03/0.03
10	0.15/0.17	0.15/0.15	0.15/0.15	0.15/0.15	0.17/0.17	0.17/0.17	0.17/0.17	0.12/0.12	0.07/0.10	-	0.00/0.00	0.00/0.00	0.05/0.02
11	0.20/0.24	0.20/0.22	0.20/0.22	0.20/0.22	0.22/0.24	0.22/0.24	0.22/0.24	0.17/0.17	0.10/0.14	0.05/0.07	-	0.02/0.00	0.08/0.03
12	0.36/0.44	0.36/0.42	0.36/0.42	0.36/0.42	0.36/0.46	0.37/0.46	0.37/0.46	0.29/0.31	0.25/0.27	0.20/0.29	0.17/0.22	-	0.10/0.14
13	0.27/0.34	0.29/0.32	0.29/0.32	0.29/0.32	0.27/0.36	0.29/0.36	0.29/0.36	0.20/0.24	0.19/0.20	0.17/0.20	0.15/0.15	0.02/0.03	-

Notes: 1—oral counting from 1 to 30; 2—one-to-one correspondence counting within 30; 3—cardinality within 30; 4—numerical literacy within 30; 5—sum (under 10) of two numbers; 6—difference (under 10) between two numbers; 7—compose and decompose number 10; 8—forward verbal counting 30 numbers from a specific number within 30; 9—backward verbal counting from a specific number within 30 to 1; 10—unknown addend (within 30); 11—unknown subtrahend (within 30); 12—unknown summand (within 30); 13—unknown minuend (within 30). Percentages on the left and right side of the slash are for boys and girls, respectively.

## Data Availability

The data presented in this study are available on request from the author. The data are not publicly available due to research ethics statements that were declared in the informed consents.
